# The Function of E-Cadherin in Stem Cell Pluripotency and Self-Renewal

**DOI:** 10.3390/genes2010229

**Published:** 2011-02-25

**Authors:** Francesca Soncin, Christopher M. Ward

**Affiliations:** Core Technology Facility, Faculty of Medical and Human Sciences, The University of Manchester, 46 Grafton Street, M13 9NT, UK; E-Mail: fsoncin@sanfordburnham.org

**Keywords:** E-cadherin, pluripotency, embryonic stem cell, induced pluripotent stem cell, iPS, ES, signaling pathways, Activin, Nodal

## Abstract

Embryonic stem (ES) and induced-pluripotent stem (iPS) cells can be grown indefinitely under appropriate conditions whilst retaining the ability to differentiate to cells representative of the three primary germ layers. Such cells have the potential to revolutionize medicine by offering treatment options for a wide range of diseases and disorders as well as providing a model system for elucidating mechanisms involved in development and disease. In recent years, evidence for the function of E-cadherin in regulating pluripotent and self-renewal signaling pathways in ES and iPS cells has emerged. In this review, we discuss the function of E-cadherin and its interacting partners in the context of development and disease. We then describe relevant literature highlighting the function of E-cadherin in establishing and maintaining pluripotent and self-renewal properties of ES and iPS cells. In addition, we present experimental data demonstrating that exposure of human ES cells to the E-cadherin neutralizing antibody SHE78.7 allows culture of these cells in the absence of FGF2-supplemented medium.

## 1. Introduction

**Figure 1:**
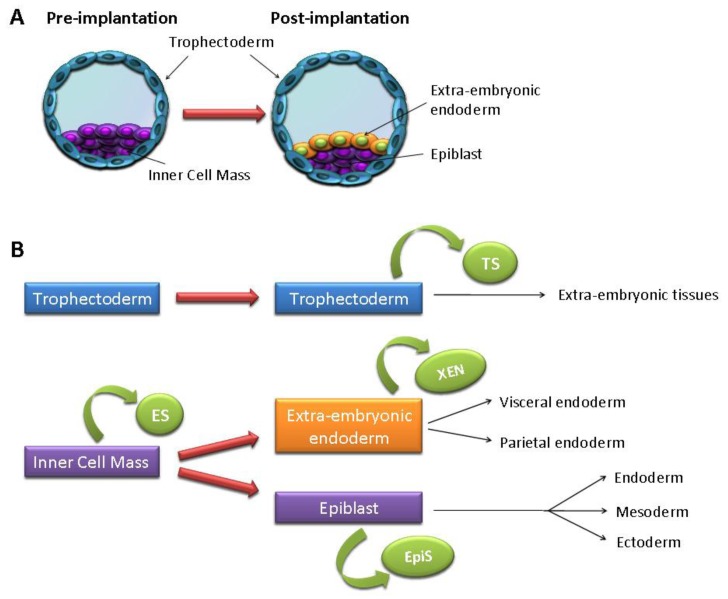
A generalized view of the derivation of stem cells from pre- and post-implantation stages. (**A**) Pre-implantation blastocysts (left) are formed by two different cell types; the external trophectoderm surrounds a cavity where the inner cell mass (ICM) is found. In post-implantation stages, the ICM differentiates into the epiblast (internal) and the extra-embryonic endoderm (in contact with the cavity); (**B**) List of cell types found in pre- and post-implantation stages and the relative stem cell lines that have been isolated and maintained *in vitro* (green). ES = Embryonic Stem cells from the ICM, TS = Trophoblast Stem cells from the trophectoderm, XEN = Extraembryonic endoderm-derived cells, EpiS = Epiblast Stem cells. The black arrows indicate the tissues that each cell type will develop into during embryogenesis.

“Stemness” can be defined as a generic state of a cell that possesses the ability to self-renew and give rise to more differentiated progeny. As a result, stemness encompasses a large range of cell types, including embryonic stem (ES) cells, germ cells, tissue-specific stem cells and cancer stem cells. In the context of ES and induced pluripotent stem (iPS) cells, stemness is characterized by the ability of a cell to differentiate to all lineages of the three primary germ layers (pluripotency) and to symmetrically divide to produce pluripotent cells (self-renewal) (Figure 1). Studies on teratocarcinomas, a rare cancer of germ cells characterized by the presence within the tumoral mass of various differentiated cell types, led to the isolation of undifferentiated pluripotent cells called embryonal carcinoma (EC) cells [1,2]. The knowledge acquired from the isolation and culture of EC cells led to the derivation of ES cells from the inner cell mass of mouse pre-implantation blastocysts in 1981 (Figure 1) [3,4]. Isolation of ES cells from various other species followed, such as pig (1990) [5], rabbit (1993) [6] and chicken (1996) [7], with the first non-human primate ES cells isolated from Rhesus monkey [8] and common marmoset [9]. The first human ES cell lines were derived in 1998 by Thomson and colleagues [10], over 15 years after mouse ES cells. Characterization of mouse (m) and human (h) ES cells has shown that, whilst derived from similar tissues, they represent unique cell types with distinct features. In 2007, two groups isolated mouse stem cells from epiblast tissues of post-implantation stages, termed Epi stem (EpiS) cells, which exhibited properties more similar to hES cells than mES cells [11,12] (Figure 1).

Recently, pluripotent cells have been isolated from different stages of mouse and human embryo development, including cleavage-stage embryos, individual blastomeres [13–15] and parthenogenic embryos [16–18]. Additionally, stem cells have been isolated from trophectoderm (trophoblast stem cells, TS) [19], extraembryonic endoderm (XEN cells) [20], primordial germ cells (germ stem (GS) cells) [21,22] (Figure 1) and various adult tissues [23]. These different stem cells represent unique cell lines with specific characteristics and distinct differentiation potential. Recently, to overcome immunological as well as ethical issues arising from the use of human embryos, Yamanaka and colleagues reprogrammed somatic cells to generate iPS cells [24,25]. These cells were obtained via forced expression of specific genes (Oct3/4, c-Myc, Sox2, Klf4) following viral transfection of adult fibroblasts. To date, various combinations of genes (e.g., Oct3/4, Sox2, Nanog and Lin28) [26] as well as different somatic cell types (e.g., liver and stomach cells [27], pancreatic β cells [28] and B cells [29]) have been successfully utilized to derive iPS cells. Whilst iPS cells provide a potentially useful alternative to ES cells for clinical therapy applications, they also allow the study of lineage specification of cells isolated from patients with genetic diseases [30].

ES and iPS cells can self-renew for prolonged periods *in vitro* whilst retaining a stable diploid karyotype (reviewed in [31]). ES cells were initially maintained in culture in the presence of non-proliferating primary mouse embryonic fibroblasts, called feeders, but considerable effort has been made to develop feeder-free culture medium and, more recently, fully-defined conditions for the culture of these cells [32]. ES cells grow as individual colonies, maintained via E-cadherin-mediated cell-cell contact, and express a panel of highly conserved epitopes of which some appear to be species-specific [33,34]. Transcription profiling studies have revealed that over 60% of genes are expressed in ES cells (compared to only 10–20% in somatic cells) and most of these are involved in signal transduction and regulation, making ES cells very responsive to the microenvironment [35,36]. Upon differentiation, pluripotent stem cells modify their gene expression resulting in a distinctive transcript expression profile dictated by lineage commitment.

**Figure 2:**
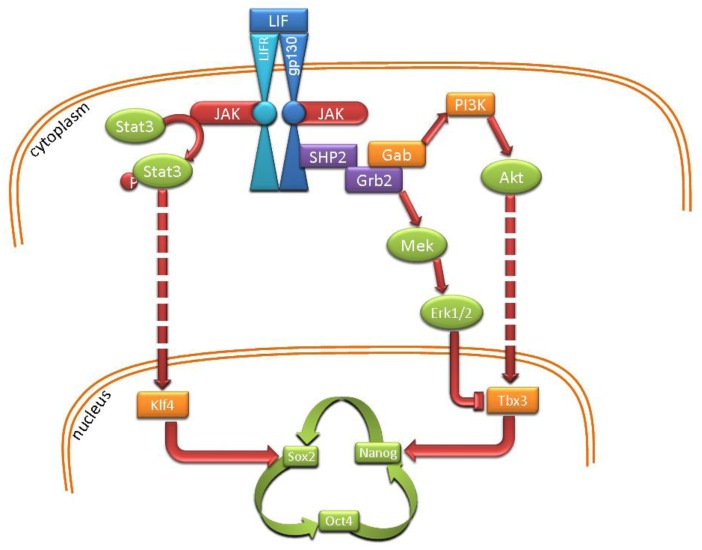
Diagrammatic representation of the pathways associated with leukemia inhibitory factor (LIF)-dependent pluripotency in mouse ES cells.

A circuitry of core genes with transcription factor activity has been identified to be essential for maintenance of the pluripotent state of ES cells. Oct3/4, Sox2 and Nanog form a key network in both mouse and human ES cells [37]. They regulate each other's expression as well as functioning as transactivators of many other genes [38–41]. Maintenance of optimal levels of these genes is fundamental for ES cell pluripotency as both up-regulation or down-regulation of individual components of the network can induce differentiation of the cells [42–44]. Besides this core group of genes (Figure 2), other molecules have emerged as important regulators of ES cell pluripotency and self-renewal, such as c-Myc (for the control of cell cycle) and Tbx3 and Klf4 for maintenance of Nanog and Sox2 expression [45,46]. Moreover, recent progress in chromatin and microRNA analysis has unveiled the role of epigenetic modifications and miRNA in regulating stem cell pluripotency and self-renewal [47–49] and the differentiation of these cells [50].

## 2. Pluripotent Signaling Pathways in ES Cells

### 2.1. Signaling Pathways in Mouse ES Cells

mES cells were initially isolated in the presence of mitotically inactivated feeder cells [31]. In 1988, leukemia inhibitory factor (LIF), a member of the interleukin-6 family of cytokines, was identified as an essential factor for maintaining mES cell pluripotency in the absence of feeder cells [51,52]. Subsequent analysis has demonstrated that binding of LIF to its receptor (LIFR) leads to dimerization of LIFR with gp130 and activation of various parallel signaling cascades (Figure 2). LIFR/gp130 dimerization activates the Janus-associated tyrosine kinases (JAK), which phosphorylate the signal transducer and activator of transcription factor 3 (Stat3) [53] (Figure 2). Phosphorylation of Stat3 has been associated with prolonged maintenance of ES cells in LIF- and serum-supplemented medium in a Nanog-independent manner [54–56]. LIF activity has also been associated with the phosphoinositol-3-kinase (PI3K) and the Grb2/MAPK (ERK mitogen-activated protein kinase) cascades [57,58]. Recently, Niwa and colleagues have shown that these three parallel cascades of the LIF signaling pathway work via separate mediators on different members of the core pluripotency network [45]. Stat3 activates Klf4 which, in turn, sustains expression of Sox2. Both PI3K and Grb2/MAPK target the transcription factor Tbx3 but with opposing effects [59,60], the former positively regulating Tbx3 via Akt signaling, which results in Nanog expression [61,62] (Figure 2). Therefore, pluripotent signaling networks in mES cells are tightly controlled via both positive and negative regulation, with small perturbations in these pathways sufficient to induce differentiation of the cells to specific lineages [45,63].

**Figure 3:**
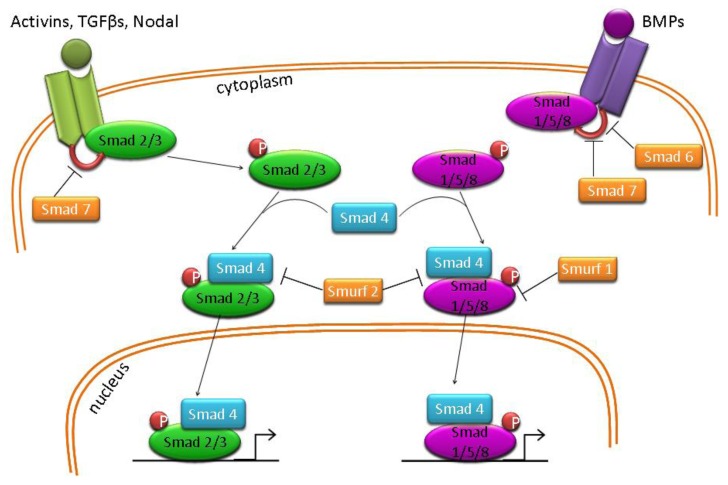
Diagrammatic representation of Activin, Nodal, TGFβ and BMP signaling pathways.

Albeit important, LIF alone is not sufficient to maintain ES cells in the absence of serum [64]. Bone morphogenetic protein 4 (BMP4), a member of the transforming growth factor beta (TGFβ) family, works in tandem with LIF in serum-free conditions to maintain ES cell pluripotency. BMP4 signals through a branch of the TGFβ cascade which involves the activation of the transduction molecules Smad1/5/8, which have been shown to activate the inhibitor of differentiation (Id) proteins (Figure 3) [64]. Ying and colleagues [64] have shown that overexpression of Id proteins allow culture of mES cells in the presence of LIF and absence of either serum or BMP4. They have subsequently demonstrated that mES cells can be cultured in the absence of LIF and BMP4 in medium supplemented with antagonists of mitogen-activated protein kinase (ERK1/2) and glycogen synthase kinase 3 (GSK3), defining a “ground state” of ES cell self-renewal, which is independent of exogenous factor supplementation [63].

### 2.2. Signaling Pathways in Human ES Cells

Human ES (hES) cells can be maintained in an undifferentiated state on various types of feeder layers, such as mitotically-inactivated mouse embryonic fibroblasts (MEFs) and human foreskin fibroblasts [10,65,66]. However, unlike their mouse counterpart, hES cell pluripotency cannot be sustained by LIF [67,68] and the BMP pathway induces trophoblast differentiation in serum-containing medium [69] or extra-embryonic specification in chemically defined medium (CDM) [70]. Instead, hES cells utilize the fibroblast growth factor (FGF) and the Activin/Nodal/TGFβ signaling cascades (Figure 3), which appear to work in cooperation at low or moderate levels of the ligands as well as influencing each other at higher concentrations [71]. In serum-containing medium, high concentrations of FGF2 sustain hES cell pluripotency via inhibition of BMP cascades [72–74] and by inducing TGFβ1 expression [75]. However, in CDM, FGF2 activity appears to act independently of BMP signaling inhibition [71]. In serum-containing media, high concentrations of Activin A is sufficient to maintain hES cells in an undifferentiated state and this effect might be partially due to induction of FGF2 expression [71,76]. In CDM, the activity of FGF2 and Activin/Nodal appear to be related and dose-dependent. For example, FGF2 alone causes neuroectoderm formation whereas a combination of high levels of Activin, BMP4 and FGF2 induces mesendoderm specification of hES cells [70]. Therefore, tight regulation of these two signaling pathways maintains hES cell pluripotency whereas small perturbations are sufficient to induce differentiation.

Recently, mouse EpiS cells, isolated from the epiblast tissue of post-implantation blastocysts, have been shown to utilize FGF and the Activin/Nodal/TGFβ signaling pathways in a similar manner to hES cells [11,12]. Although these cells integrate poorly into blastocysts to form chimeras, they exhibit core pluripotent marker expression and self-renewal properties typical of ES cells. However, whilst expression of the basic pluripotent network proteins Oct3/4, Sox2 and Nanog are maintained, mouse EpiS cells exhibit a transcriptional signature more similar to hES than mES cells [12].

### 2.3. Role of the Wnt Pathway in ES Cell Pluripotency and Self-Renewal

The role of the canonical Wnt pathway in regulating ES cell pluripotency and self-renewal remains a question of debate. Various groups have described that sustained activation of the Wnt pathway can maintain pluripotency in both mouse and human ES cells [77–80]. In 2008, Chou and colleagues [81] described the isolation of cells from mouse blastocysts using a chemically defined medium containing FGF2, Activin A, BIO (a GSK3β inhibitor which mimics canonical Wnt activation by stabilizing active β-catenin protein levels) and a LIF inhibitor. These cells, termed FABS cells, showed similar gene expression profiles to EpiS cells, although with unique features. These results suggest a role of the canonical Wnt pathway in the maintenance of ES cell pluripotency. However, very low levels of Wnt activity are detected in ES cells cultured in either feeder or feeder-free conditions [77,78], suggesting that this pathway is not essential for maintaining pluripotency in these cells. Moreover, Wnt activity has also been associated with induction of cell differentiation in various cellular environments (e.g., bone morphogenesis and muscle specification), thus posing the question of how the canonical Wnt pathway can maintain pluripotency of ES cells and, at the same time, induce differentiation [81,82]. Dravid and colleagues have attempted to resolve this apparent conflict by suggesting a role for the Wnt pathway in the induction of ES cell proliferation (self-renewal) [83]. They suggest that the canonical Wnt pathway might not affect ES cell pluripotency or differentiation *per se* but, by sustaining cell proliferation, accelerate the outcome determined by other factors within the environment. Sineva *et al.* [84] have recently demonstrated that culture of mES cells in the GSK3 inhibitor BIO accentuates both E-cadherin/β-catenin interaction and TCF/β-catenin transactivation and is associated with decreased proliferation of the cells. In contrast, Doble and colleagues [85,86] showed that deletion of all GSK3 isoforms in mES cells caused increased β-catenin protein levels and β-catenin/TCF trans-activation activity with no loss of ES pluripotency and no changes in proliferation rates compared to wildtype (wt) ES cells. However, GSK3 double-knock-out ES cells showed impaired differentiation abilities. In addition, we have described the culture of β-catenin null mouse ES cells in serum-containing media supplemented with LIF [87], suggesting that β-catenin does not play a role in the core pluripotent signaling network of mES cells. Therefore, the exact role of β-catenin in maintaining ES cell pluripotency and self-renewal remains unclear.

## 3. E-cadherin and Its Interacting Proteins

Cell adhesion is essential for embryonic development [88,89] and cell-cell interaction has been shown to influence adult stem cell differentiation [90,91]. ES cells grow in individual colonies exhibiting cell-cell adhesion mediated by various complexes belonging to adherens junctions (AJs), tight junctions, desmosomes and gap junctions [31]. AJs are formed by E-cadherin and associated catenin proteins and are necessary for the establishment and maintenance of cell-cell contact [88].

### 3.1. E-Cadherin

**Figure 4:**
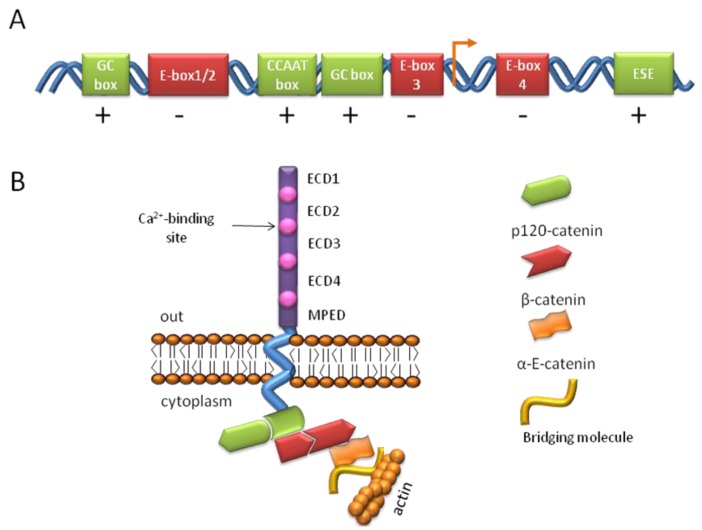
Diagrammatic representation of the E-cadherin promoter and protein. (**A**) Promoter region of the Cdh1 gene encoding for E-cadherin. The figure shows both positive (GC boxes and CCAAT boxes) and negative (E boxes) regulatory elements. Note the location of E-box 4 after the transcription initiation site (orange arrow) and the presence of an epithelial specific enhancer (ESE) located within an unusually large intron 2. (**B**) Diagrammatic representation of the E-cadherin/catenin complex anchored to the actin cytoskeleton. The extracellular region of E-cadherin contains four extracellular cadherin domains (ECD) and an atypical membrane proximal domain (MPED). Calcium ion-binding sites are located between ECDs and are necessary for cell adhesion mediation. β-Catenin and p120-catenin bind specific regions within the cytoplasmic domain of E-cadherin. α-E-catenin might directly anchor the complex to the actin cytoskeleton by binding with F-actin and β-catenin or indirectly through an additional bridging molecule (e.g., EPLIN). ECD = extracellular cadherin domain; MPED = Membrane Proximal Extracellular Domain.

Cadherins are a large family of transmembrane or membrane-associated glycoproteins characterized by the presence of multiple repeats of a specific extracellular cadherin domain (ECD) [92]. Among the five major sub-families of cadherins, E-cadherin belongs to the type-I cadherin group and is generally considered the prototype of all cadherins due to its early identification and thorough characterization [93]. The E-cadherin gene (Cdh1) is located on chromosome 16 in human and chromosome 8 in mouse and are similarly organized into exon-intron tandems [94]. The spatio-temporal regulation of E-cadherin in embryonic development is tightly controlled by a complex promoter region containing multiple activating and silencing sequences [89,93] (Figure 4A). Positive regulators of E-cadherin expression include a CCAAT box, GC boxes [95] and a conserved epithelial specific enhancer (ESE) of transcription within intron 2, which regulates E-cadherin expression during embryogenesis. E-cadherin knock-out mice die before implantation as a consequence of lack of trophectoderm formation, demonstrating the critical role this protein plays during development [88,96-98]. E-cadherin expression is silenced by numerous transcription factors that bind various E-pal boxes within the E-cadherin promoter region (Figure 4A). Among these negative regulating binding factors are Snail, Slug, E12/E47 and the zing finger factors δEF1/ZEB1 and SIP1/ZEB2 [93]. These repressors of E-cadherin expression appear to act downstream of various signaling pathways, such as TGFβ, FGF, nuclear factor κB (NFκB) and integrin cascades.

E-cadherin is a single-pass transmembrane glycoprotein with an extracellular region containing five tandemly organized domains, four of which are typical extracellular cadherin domains (ECD), whilst the fifth is defined as the Membrane Proximal Extracellular Domain (MPED) and consists of four conserved cysteines essential for E-cadherin function (Figure 4B) [93]. The ECDs are interspersed by Ca^2+^-binding regions which are essential for cell-cell contact. The cytoplasmic domain of E-cadherin contains binding regions for β-catenin/plakoglobin and p120-catenin (Figure 4B) as well as various regulatory elements (e.g., phosphorylation sites and regions recognized by the degradation machinery of the cell). In general, catenins allow the stabilization of the cytoplasmic cell adhesion complex, protecting it from degradation and maintaining anchorage of E-cadherin to the actin cytoskeleton.

E-cadherin interacts in a homophilic manner (preferential binding to another E-cadherin molecule) as well as in a homotypic fashion (binding to the same cell type) [99]. However, the exact topography of this interaction and the mechanisms that allow such exclusivity in the presence of other cadherins with similar extracellular domains remain unclear. E-cadherin appears to form *cis*-homophilic dimers with molecules on the same cell and *trans*-homophilic dimers with molecules on neighboring cells. The first ECD is essential for cell adhesion initiation but various hypotheses suggest stable cell adhesion might involve one or more of the other ECDs within the extracellular region of E-cadherin. For example, the antibody DECMA-1, which inhibits E-cadherin-mediated cell-cell contact, recognizes an epitope in ECD4 and MPED [100,101]. Recent findings have highlighted the presence of a hydrophilic pocket within ECD1 with which the *N*-terminal region of a neighboring E-cadherin molecule may interact [102]. The conserved Histidine-Alanine-Valine (HAV) tripeptide in ECD1 has been shown to be important for *trans*-homodimerization of E-cadherin but its role is not fully clarified [103,104].

**Figure 5:**
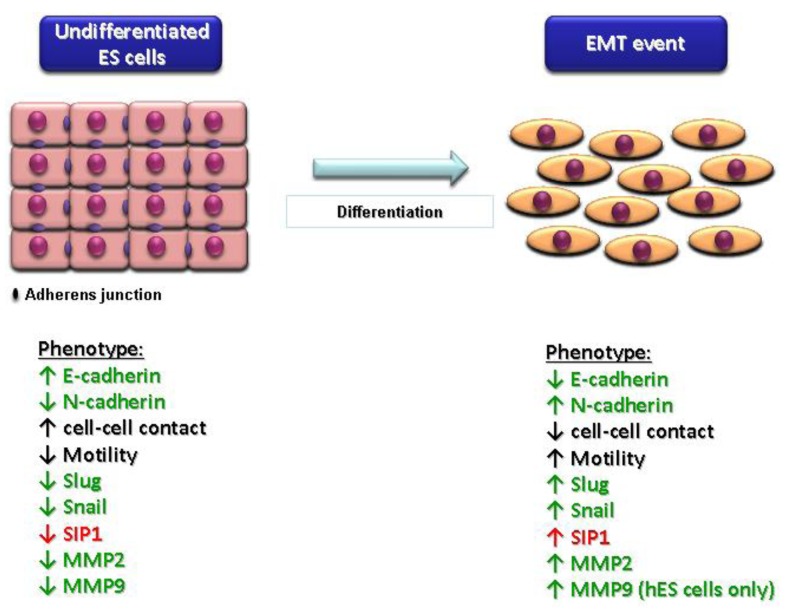
Diagrammatic representation of the transcriptional and translational events associated with epithelial-mesenchymal transition during mouse and human ES cell differentiation. Undifferentiated ES cells exhibit E-cadherin-mediated cell-cell contact and this is associated with low levels of N-cadherin, E-cadherin repressors (Slug, Snail and SIP1) and matrix metalloproteinases (MMPs) [112,113]. Upon induction of ES cell differentiation, E-cadherin protein is rapidly lost from the cell surface and this is associated with increased N-cadherin, E-cadherin repressor (Slug, Snail and SIP1) and MMP expression [112,113]. Green denotes changes in both transcripts and protein; red denotes changes in transcripts only.

Regulation of E-cadherin expression is fundamental to vertebrate development [96]. E-cadherin is expressed in most adult epithelial tissues and has been shown to be a potent tumor invasion suppressor [105,106–108]. Loss of E-cadherin is associated with epithelial-mesenchymal transition (EMT), which is a crucial process in various stages of embryogenesis, tissue repair and tumor invasion [109,110]. EMT is characterized by loss of E-cadherin mediated cell-cell contact and acquisition of a more motile phenotype. Changes occurring during EMT comprise morphological modifications, altered cellular adhesion and motility, acquisition of anterior-posterior polarity, resistance to apoptosis and upregulation of matrix metalloproteinases [111]. EMT is characterized by a switch between E-cadherin, via upregulation of E-cadherin repressors (e.g., Slug and Snail), and a less adhesive cadherin, such as N-cadherin. During embryogenesis, regulation of E-cadherin and EMT is crucial for proper cell sorting, cell movement, polarity maintenance and barrier tissue formation. Our group has previously shown that ES cell differentiation is associated with an EMT-like event [112,113], and this is summarized in Figure 5. Loss of E-cadherin has been observed in many tumors of epithelial origin (e.g., gastric, gynecological and breast) and is often associated with EMT and poorer patient prognosis.

### 3.2. β-Catenin

β-catenin is a 90 kDa cytoplasmic protein characterized by the presence of 12 Armadillo repeats. It binds a specific region within the cytoplasmic domain of E-cadherin (Figure 4B) and this binding is essential for cell-cell adhesion, as shown by mutagenesis studies of the E-cadherin protein [114]. β-catenin knock-out mice are nonviable and embryos die around gastrulation [115]. Plakoglobin appears to be able to substitute for β-catenin loss at adherens junctions during early stages of embryo development in β-catenin null mice, although β-catenin is subsequently necessary for the anterior-posterior axis formation. β-catenin null ES cells exhibit decreased cell-cell contact, similar to that observed in E-cadherin null ES cells [116,117]. Besides its structural role, β-catenin can also function as a transcriptional regulator in response to specific signals, in particular, as a molecular effector of canonical Wnt signaling [118]. In the nucleus, β-catenin interacts with the TCF/LEF complex and regulates expression of numerous Wnt target genes.

### 3.3. α-E-Catenin

α-E-catenin (epithelial) is a member of the atypical α-catenin family, which also comprises α-N-catenin (neuronal) and α-T-catenin (heart). α-E-catenin exhibits a structure similar to vinculin and it considerably differs from other catenins in that it lacks Armadillo repeats [119]. Genetic studies have shown the importance of α-E-catenin in embryo development and ES cells, mainly due to its role in cell adhesion [120,121]. For example, inhibition of α-E-catenin protein expression in ES cells, via gene trap mutation, resulted in a phenotype similar to E-cadherin knock-out mice, suggesting that the main function of α-E-catenin is to maintain and stabilize E-cadherin-mediated cell-cell contact [120]. Recent evidence suggests that an intermediate protein, such as epithelial protein lost in neoplasm (EPLIN) [122], may link β-catenin-bound α-E-catenin to the actin cytoskeleton (Figure 4B).

### 3.4. p120-Catenin

p120-Catenin is a typical catenin member containing 10 Armadillo repeats [123]. It binds a specific site within the cytoplasmic domain of E-cadherin and appears to function to prevent degradation of E-cadherin protein by inhibiting endocytic membrane trafficking [124–127]. It also promotes recycling of E-cadherin to the cell surface via interaction with kinesin motors [128]. Similar to α- and β-catenin, there is increasing evidence to suggest that p120-catenin might play additional roles besides cell adhesion [129]. For example, within the cytoplasm, p120-catenin modulates the opposing activity of the Rho and Rac families of GTPases, thus contributing to cytoskeletal organization [130]. p120-Catenin can also translocate into the nucleus where it may interact with the transcription factor Kaiso, resulting in gene transactivation [131–133]. The Kaiso/p120-catenin pathway seems to overlap, at least partially, with the TCF/β-catenin complex activity in the regulation of Wnt target genes [134,135]. Many of these p120-catenin activities have been associated with increased cellular proliferation and altered cell cycle in cancer cells [134,136].

## 4. The Function of E-Cadherin in ES and iPS Cell Pluripotency and Self-Renewal

In recent years, evidence for the function of E-cadherin in regulating pluripotent and self-renewal signaling pathways in stem cells has emerged. Below we discuss some of the relevant literature describing the function of E-cadherin in human and mouse pluripotent stem cells.

### 4.1. E-cadherin Regulates Localization of Cell Surface Molecules in ES Cells

We and others have previously shown that loss of E-cadherin in ES cells induces major changes in cellular architecture and localization of plasma membrane-associated proteins. For example, abrogation of E-cadherin in ES cells results in altered actin cytoskeleton arrangement and induction of cell polarization [112,113]. Furthermore, E-cadherin expression in ES cells functions to inhibit cell surface localization of the 5T4 oncofetal antigen, a pro-migratory factor that is associated with poorer clinical outcome in ovarian, gastric and colorectal cancers [137–140]. Therefore, the specific cellular architecture induced by E-cadherin-mediated cell-cell contact is likely to facilitate correct localization of plasma membrane proteins. This is supported by observations that expression of Eph receptors and ephrins are differentially regulated by E-cadherin in ES cells [141]. In addition, our unpublished data suggests a role for E-cadherin in regulating correct plasma membrane localization of a range of proteoglycans in mES cells. Therefore, as well as its role in maintaining epithelial integrity, E-cadherin mediated cell-cell contact is critical for the correct presentation of a range of molecules at the cell surface of ES cells.

### 4.2. E-Cadherin Expression Regulates Signaling Pathways in Pluripotent Cells

FABS cells [81], isolated from mouse blastocysts in defined culture media containing FGF2, Activin A, BIO and LIF inhibitor, expressed markers of pluripotency although they were unable to form teratomas and failed to expand when grown in suspension. Culture of FABSCs in LIF/BMP-supplemented medium for seven days resulted in restoration of chimera forming ability and this was associated with increased expression of E-cadherin. In addition, abrogation of E-cadherin expression in these cells resulted in their differentiation. This data demonstrated that both the culture growth factor environment and cell-cell interaction play a critical role in defining specific stem cell pluripotent signaling pathways.

Whilst E-cadherin^−/−^ ES cells maintain an undifferentiated phenotype in media supplemented with fetal bovine serum (FBS) and LIF [113], we have demonstrated that these cells do not require LIF under these conditions [87]. Instead, E-cadherin^−/−^ ES cells maintain pluripotency via Activin/Nodal signaling and optimal self-renewal is achieved via FGF2. Therefore, E-cadherin functions in mES cells to positively regulate LIF/BMP-dependent pluripotency. The ability of E-cadherin^−/−^ ES cells to maintain pluripotency in FBS- and LIF-supplemented medium reflects the presence of Activin, Nodal and FGF2 within ES cell-screened serum. E-cadherin^−/−^ ES cells maintain pluripotent marker expression in serum-free medium supplemented with Activin, Nodal and FGF2 and exposure of these cells to the Activin-like kinase receptors (Alks)-4, -5 and -7 inhibitor (SB431542) induces differentiation of the cells [87]. Using mutant E-cadherin expression vectors, we determined that the β-catenin binding region of E-cadherin is essential for LIF/BMP-mediated pluripotency in ES cells. In addition, reversible Activin/Nodal-dependent pluripotency could be induced in wild type (wt)ES cells by their treatment with an E-cadherin homodimerization-inhibiting peptide, CHAVC. Furthermore, E-cadherin^−/−^ ES cells can also be maintained in an undifferentiated state in serum-free medium supplemented with LIF/BMP, suggesting that these cells exhibit altered hierarchy of pluripotent regulating pathways [63]. Interestingly, we also demonstrated that β-catenin^−/−^ ES cells maintain pluripotency via Activin/Nodal and self-renewal via FGF2, demonstrating that a functional E-cadherin/β-catenin complex is critical for LIF/BMP-mediated pluripotency in mouse ES cells. This was the first demonstration of multiple pluripotent signaling networks existing in ES cells and that the hierarchical activity of these pluripotent states is determined by the E-cadherin/β-catenin complex.

**Figure 6:**
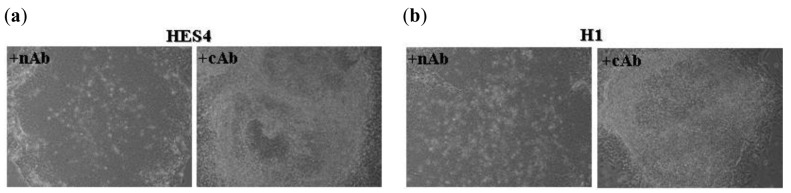

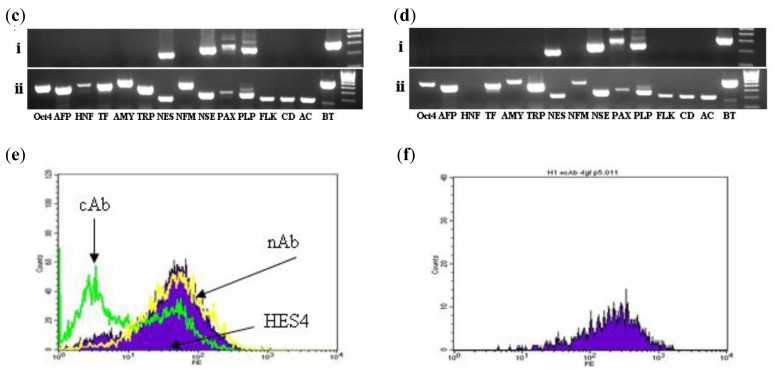
Culture of hES cells in the presence of E-cadherin neutralizing antibody SHE78.7 allows their culture in the absence of FGF2. HES4 and H1 human ES cell lines were cultured in the presence of a minimal fibroblast feeder layer (approximately 1000 cells/dish) in the absence of FGF2 in serum replacement medium in the presence of cAb or nAb (0.5 μL/mL of media of 0.5 mg/mL stock solution). (**a**) Phase contrast microscopy of HES4 ES cells cultured in control antibody (cAb) or E-cadherin neutralizing antibody SHE78.7 (nAb) after 2 passages in the absence of FGF-2. Note that nAb cells exhibited normal colony morphology whereas cAb treated cells differentiated; (**b**) Phase contrast microscopy of H1 ES cells cultured in control antibody (cAb) or E-cadherin neutralizing antibody SHE78.7 (nAb) after 2 passages in the absence of FGF-2. Note that nAb cells exhibited normal colony morphology whereas cAb treated cells differentiated; (**c**) (i) HES4 ES cell colonies were cultured in nAb (0.5 μL/mL of media of 0.5 mg/mL stock solution) in the presence of a minimal fibroblast feeder layer in the absence of FGF2 in serum replacement medium for 10 passages (approximately 90 days) and assessed for expression of transcripts associated with pluripotency and various lineage markers as previously described [142]. Note that the transcript profile expression is consistent with that observed for undifferentiated HES4 ES cells (as described in Ward *et al.* [142]). (ii) HES4 ES cell colonies described above were allowed to overgrow in the culture plates (*i.e.*, no passaging) in normal ES cell culture medium (*i.e.*, +FGF2) for 20 days to induce differentiation of the cells and assessed for expression of transcripts associated with pluripotency and various lineage markers (as described in [142]). Note that markers of differentiation expressed following differentiation of the cells included all three germ layers (endoderm-HNF, TF, AMY; mesoderm-FLK, CD34, AC133; ectoderm-NES, NFM, NSE, PAX and PLP) and extra-embryonic visceral endoderm (AFP); (**d**) RT-PCR analysis of (i) undifferentiated and (ii) differentiated H1 ES cells as described in (c). Oct-4 (OCT); α-fetoprotein (AFP); hepatocyte nuclear factor (HNF); nestin (NES); neurofilament middle chain (NFM); neuron-specific enolase (NSE); Pax-6 (PAX); proteolipid protein (PLP); amylase (AMY); α1-antitrypsin (TRP); Flk-1 (Flk); CD34 (CD); AC133 (AC); Transferrin (Tf); β-tubulin (BT); alpha-fetal protein (AFP); (**e**) Cell surface expression of the pluripotent marker Tra-1-60 was assessed on cAb and nAb treated HES4 ES cells (HES4) after 3 passages in the absence of FGF2 and HES4 ES cells cultured under normal conditions (HES4) on a fibroblast feeder layer containing FGF2 by fluorescent flow cytometry. Note that nAb-treated cells exhibited similar expression of Tra-1-60 compared to HES4 cells cultured under normal conditions; (**f**) Cell surface expression of the pluripotent marker Tra-1-60 was assessed on nAb treated HES4 ES cells (all cAb treated cells died) after 5 passages in the absence of FGF2. Note that >99% of the nAb treated cells exhibited Tra-1-60 expression.

We have previously demonstrated that inhibition of E-cadherin-mediated cell-cell contact in hES cells using a neutralizing antibody (nAb) does not induce differentiation of these cells [112], although it does decrease their proliferative capacity. Titration of nAb onto hES cells such that cell-cell contact and proliferation are unaffected allowed the prolonged culture of the cells in the absence of FGF2 (Figure 6). After two passages in the presence of nAb and absence of FGF2, both HES4 (Figure 6a) and H1 (Figure 6b) ES cells exhibited characteristic colony morphology whereas cAb treated colonies were mostly differentiated. Cell surface expression of the pluripotent marker Tra-1-60 was assessed on cAb and nAb treated HES4 hES cells after three passages in the absence of FGF2 (Figure 6e). nAb-treated HES4 ES cells exhibited similar expression of Tra-1-60 compared to HES4 cells cultured under normal conditions. By contrast, cAb-treated HES4 hES cells exhibited significantly decreased expression of Tra-1-60. Similarly, H1 hES cells treated with nAb for five passages in the absence of FGF2 exhibited high levels of Tra-1-60 expression (Figure 6f) whereas all cAb treated cells had died. Following culture of H1 and HES4 ES cells for 10 passages (approximately 90 days) in the presence of nAb and absence of FGF2, RT-PCR analysis was performed to assess expression of transcripts associated with pluripotency (Oct4) and various lineage markers (Figure 6c(i) and 6d(i)). Both cell lines exhibited an undifferentiated transcript profile consistent with our previous observations [142]. To determine the differentative potential of hES cells cultured for 10 passages in the presence of nAb and absence of FGF2, we allowed the cells to overgrow in the culture plates (*i.e.*, no passaging) in normal ES cell culture medium (*i.e.*, +FGF2) for 20 days to induce differentiation and assessed the cells for expression of lineage specific transcript markers (Figure 6c(ii) and 6d(ii)). Both hES cell lines expressed markers of differentiation, demonstrating that the cells maintained the ability to differentiate into cells representative of the three primary germ layers. Whilst the exact mechanism for self-renewal of human ES cells in the absence of FGF2 is unclear (for example, it may be due to exogenous FGF2 expression induced by the nAb) it does illustrate that E-cadherin functions in both mouse and human ES cells to positively regulate pluripotent signaling pathways.

### 4.3. Manipulation of Culture Surfaces with E-Cadherin Fusion Proteins

Nagaoka *et al.* [143], have demonstrated that culture of mES cells on plates coated with an E-cadherin-Fc fusion protein exhibited decreased cell-cell adhesion and failed to form characteristic ES cell colonies. However, these cells maintained expression of pluripotent markers and were able to differentiate into cells of the three primary germs layers, demonstrating that pluripotency was unaffected in these cells. In addition, ES cells cultured on the E-cadherin-Fc fusion protein plates exhibited increased proliferation and decreased dependence on LIF, although removal of LIF from the cells induced differentiation. Similar results were also obtained for human ES and iPS cells using human E-cadherin-Fc fusion protein-coated plates [144]. Culture of hES or iPS cells in mTeSR1 medium on E-cadherin-Fc fusion protein-coated plates resulted in maintenance of pluripotent marker expression, normal proliferation rates and, after >60 days in culture, no abnormal karyotype was observed. In addition, these cells were able to form embryoid bodies which expressed lineage-specific gene transcripts and teratomas generated from these cells exhibited evidence of differentiation to the three primary germ layers. Whilst the plating efficiency of disaggregated hES cells on E-cadherin-Fc fusion protein plates was decreased compared to that of matrigel-treated plates [144], this was found to be a result of proteolytic degradation of E-cadherin on the surface of the cells by the use of Accutase cell dissociation buffer. Where enzyme-free dissociation buffer was used, hES cell plating efficiency was equivalent to that observed on matrigel-treated plates. These results demonstrate that E-cadherin-Fc coated culture plates may provide a useful method for the culture of hES and iPS cells on defined substratum. Whilst the pluripotent signaling pathways were not investigated in detail in these studies, it is possible that E-cadherin-Fc coated plates may alter dependence of ES cells to exogenous factors.

### 4.4. E-Cadherin Expression in Feeder Cells Can Enhance Maintenance of ES Cell Pluripotency

Horie *et al.* [145] have demonstrated that forced expression of E-cadherin in mouse STO and NIH3T3 cells, which were utilized as a feeder layer for mES cells, resulted in improved expression of pluripotent markers in mES cells compared to feeder cells lacking E-cadherin expression. For example, mES cells cultured on untransfected STO and NIH3T3 cells exhibited low levels of expression of the pluripotent markers Oct3/4, Nanog and Rex-1. By contrast, mES cells cultured on STO and NIH3T3 cells transfected with E-cadherin cDNA resulted in expression of the pluripotent markers comparable to ES cells cultured on primary MEFs. In addition, colony forming ability was increased in mES cells cultured on E-cadherin expressing STO or NIH3T3 feeder cells compared to untransfected cells. Culture of mES cells in conditioned medium derived from E-cadherin expressing STO or NIH3T3 feeder cells did not enhance expression of pluripotent markers, suggesting that direct contact between the ES cells and feeder layer, rather than secreted soluble factors, was a contributing factor to increased pluripotent marker expression and colony forming ability. Subsequently, the authors [145] showed that forced cell-cell contact between mES cells and the feeder cells, using magnetic interaction, increased pluripotent marker expression and colony forming ability in mES cells growing on untransfected STO or NIH3T3 cells. Whilst the precise mechanism for the ability of E-cadherin-expressing feeder cells to support the pluripotent state and self-renewal of mES cells is unclear, Horie and colleagues suggested that it may reflect enhanced signaling via either direct interaction between the cells or via the extracellular matrix.

### 4.5. Inhibition of E-Cadherin Expression Allows Culture of mES Cells in Shake Flasks

A fundamental requirement for the exploitation of ES cells in regenerative medicine is the ability to reproducibly derive sufficient numbers of cells of a consistent quality in a cost-effective manner. Adherent methods for ES cell culture are disadvantaged in that they result in heterogenous static conditions, leading to batch-to-batch variation, and are costly and labor intensive. Bioreactor culture of ES cells represents a useful tool since the method provides a scaleable, non-intensive and relatively homogenous high cell volume density microenvironment which can be easily monitored. Fok and Zandstra [146] demonstrated that E-cadherin protein is the cause of aggregation of mouse ES cells in suspension bioreactor culture and concluded that expression of E-cadherin protein is required to maintain viability of ES cells in bioreactor culture. In addition, Dang *et al.* [147] suggested that the use of an E-cadherin blocking antibody in bioreactor culture could adversely affect cell differentiation due to the importance of E-cadherin in embryogenesis. Despite these concerns, we have recently demonstrated that mES cells can be cultured as a near-single cell suspension in scalable shake flasks over prolonged periods without additional media supplements [148]. Wild-type D3 mES cells treated with an E-cadherin neutralizing Ab (DECMA1; EcadAb) exhibited doubling times of 15.6 ± 4.7 h and 16 ± 0.9 h mean-fold increase in viable cell numbers over 48 h. Furthermore, EcadAb ES cells propagated as a dispersed cell suspension for 15 day maintained expression of pluripotent markers, exhibited a normal karyotype and high viability and were able to differentiate to cells representative of the three primary germ layers [148]. Therefore, inhibition of E-cadherin expression in mouse ES cells represents a useful method for the cost-effective suspension culture of these cells, significantly decreasing the requirement for technical input and plastic consumables associated with adherent culture methods.

### 4.6. E-Cadherin Expression Enhances hES Cell Colony Formation and Self-Renewal

The Rho-associated kinase (ROCK) inhibitor Y-27632 has been demonstrated to increase survival of dissociated hES cells [149]. Li and colleagues [150] observed that whilst Y-27632 increased clonogenicity of hES cells, this was likely to reflect increased motility of the cells resulting in ∼60% of colonies consisting of reaggregated cells rather than colonies originating from a single founding cell. They also utilized a doxycycline-inducible E-cadherin expression vector in hES cells to assess the role of E-cadherin in promoting cell survival. They found that E-cadherin expression was associated with expression of the apoptotic inhibitory gene Bcl-XL and inhibition of the pro-apoptotic gene Caspase-3, increasing clonogenicity of hES cells up to 20-fold [150]. Furthermore, they observed that >98% of individual hES cells which failed to maintain E-cadherin expression following single cell dissociation exhibited cell death or differentiation within 72 h of seeding.

Xu and colleagues [151] identified two small molecules, Thiazovivin (Tzv) and Tyrintegin (Ptn), which increased survival of single hES cells over 30-fold whilst having little impact on proliferation. They observed that the compounds increased the level of HUTS-21 binding in hES cells, suggesting that they may function by stimulating integrin activity following interaction of cells with extracellular matrix (ECM). Small molecule Tzv was also found to promote suspended aggregate formation of hES cells by stabilizing cell surface E-cadherin expression, probably by inhibiting endocytosis of the protein. The authors also found that hES cell survival could be increased by seeding cells onto plates coated with an E-cadherin-Fc protein. They concluded that cleavage of E-cadherin itself following dissociation of hES cell colonies is not the direct cause of cell death but rather it is the destabilization of E-cadherin at the cell membrane leading to inhibition of cell-cell contact and apoptosis. ROCK was identified as a direct target of small molecule Tzv and inhibition of this pathway by Tzv is likely to be responsible for increasing hES cell survival by stabilizing E-cadherin protein at the plasma membrane. Interestingly, Xu *et al.* [151] demonstrated that treatment of hES cells with the mitogen-activated protein kinase/extracellular signal-regulated kinase kinase (MEK) inhibitor PD0325901 and p38 inhibitor SB203580 allowed their self-renewal under mES cell culture conditions (*i.e*., with LIF), and that abrogation of E-cadherin with a nAb significantly decreased proliferation of the cells under these conditions. They concluded that E-cadherin regulation and expression levels in human and mouse ES cells is likely to reflect the different culture conditions required for these cell lines. This is supported by our observations in mouse and human ES cells where aberrant expression of E-cadherin can alter cellular dependence on exogenous factors. Li *et al.* [152] have also demonstrated that functional interactions between small GTPase Rap1 and E-cadherin are responsible for regulating self-renewal of hES cells. They demonstrated that inhibition of Rap1 suppresses colony formation and self-renewal of hES cells by affecting the endocytic recycling pathway associated with formation and maintenance of E-cadherin-mediated cell-cell adhesion. They also observed that disruption of E-cadherin-mediated cell-cell contact induces degradation of Rap1, which may reflect our observations of decreased proliferation, although not pluripotency, of hES cells treated with an E-cadherin nAb [112].

### 4.7. E-Cadherin Expression Enhances iPS Cell Derivation

It has recently been demonstrated that a mesenchymal-epithelial transition (MET) event is required for nuclear reprogramming of mouse fibroblasts to iPS cells [153]. The authors showed that generation of iPS cells from mouse fibroblasts required suppression of pro-EMT signals and activation of an epithelial program within the cells. They observed that Sox2/Oct3/4 suppressed the E-cadherin repressor Snail, c-myc downregulated TGFβ1 and TGFβR2 and that Klf4 induced E-cadherin expression. In addition, when MET was blocked in the reprogramming of fibroblasts, iPS cell formation was impaired, and this was also observed in iPS cell formation using epithelial cells in which EMT was blocked. Low levels of E-cadherin protein expression in iPS cells was associated with decreased chimaera-forming efficiency, as observed in FABSCs [81], with the authors suggesting a state of “near pluripotency” of the cells. Low chimera forming efficiency of these cells is likely to reflect inefficient incorporation of the cells within the ICM, rather than being a true lack of pluripotency of the cells [81].

Chen *et al.* [154] isolated two small molecules (Apigenin and Luteolin) that enhanced E-cadherin expression and showed that these could significantly improve iPS cell derivation from mouse embryonic fibroblasts (MEFs). They further showed that forced expression of human E-cadherin in MEFs resulted in a four-fold increased isolation of iPS cells when compared to iPS-inducing factors alone (Oct3/4, Sox2, Klf4 and c-Myc). When viewed in the context of observations by Li *et al.* [150], it is possible that enhanced iPS cell derivation upon forced expression of E-cadherin is a result of increased cell-ECM interactions. Inhibition of E-cadherin, using RNAi or an inhibiting peptide, during the iPS transformation process also resulted in decreased iPS cell derivation [154]. Furthermore, they demonstrated that the β-catenin binding domain of E-cadherin was not required for optimal iPS cell derivation and that absence of the entire cytoplasmic region of E-cadherin only partially inhibited this process. In contrast, mutation of Trp2 in the extracellular region of E-cadherin abolished E-cadherin-mediated iPS reprogramming efficiency. Whilst Chen *et al.* incorrectly stated that “to maintain colony morphology has long been proposed to be the only known function of E-cadherin in ES cells” (for example, both ourselves [87] and Chou *et al.* [81] had already demonstrated a function for E-cadherin in altered cell signaling), their results do suggest that the mechanism of enhanced iPS cell-derivation via E-cadherin expression may be different to that for maintaining pluripotency in ES cells. For example, we have shown that the E-cadherin/β-catenin complex is necessary for LIF/BMP-dependent pluripotency in mES cells. Since the E-cadherin/β-catenin complex is required for cell-cell contact, these results suggest that the HAV domain within the extracellular region of E-cadherin may function to promote cellular-ECM interactions, thereby increasing clonogenicity of the cells. ES cell-ECM interactions are not required for mES cell self-renewal since we have shown that abrogation of cell-cell contact in these cells using the DECMA-1 nAb allows prolonged single cell suspension culture of pluripotent cells in shaker flasks [148]. Therefore, E-cadherin expression in ES cells may function as both a regulator of pluripotent signaling pathways as well as an enhancer of ES cell-ECM interactions to aid cell survival.

## 5. Materials and Methods

### 5.1. Human ES Cell Culture in Normal Medium

HES4 hES cells [155] and H1 hES cells [10] were cultured in tissue organ culture dishes (BD Falcon, Bedford MA, USA) coated with 0.1% gelatin and 1 × 10^5^ irradiated 129 mouse embryonic fibroblast feeder cells per dish. Cells were grown in DMEM + F12 mix media supplemented with 20% serum replacement (synthetic serum; Invitrogen Corp.), L-glutamine (1 mM), 2-mercaptoethanol (50 μM), NEAA (100×, 1:100 dilution) and bFGF (FGF2; 0.2 μg/mL in 0.1% BSA) (all Invitrogen), as described by Thomson *et al.* [10] and incubated at 37 °C/5% CO_2_. The media was changed daily. Cells were passaged after 7–10 days by gently cutting and teasing the morphologically undifferentiated cells using a yellow pipette tip and transferring colony pieces to a fresh culture dish.

### 5.2. Human ES Cell Culture in the Presence of E-Cadherin Neutralizing Antibody SHE78.7 in the Presence or Absence of FGF2

HES4 hES cells and H1 hES cells were cultured in tissue organ culture dishes (BD Falcon, Bedford MA, USA) coated with 0.1% gelatin and 1 × 10^3^ irradiated 129 mouse embryonic fibroblast feeder cells per dish. Cells were grown in DMEM + F12 mix media supplemented with 20% serum replacement (synthetic serum; Invitrogen Corp.), L-glutamine (1 mM), 2-mercaptoethanol (50 μM) and NEAA (100×, 1:100 dilution) and presence or absence of bFGF (FGF2; 0.2 μg/mL in 0.1% BSA) (all Invitrogen) as described by Thomson *et al.* [10] and incubated at 37 °C/5% CO_2_ in the presence of a control antibody (Mouse IgG_2a_; Invitrogen Corp) or E-cadherin neutralizing antibody SHE78.7 (0.5 μL/mL of media of a stock 0.5 mg/mL solution), the latter of which inhibits E-cadherin mediated cell-cell contact by binding the CD1 region of the extracellular domain. The media was changed daily. Cells were passaged after 7–10 days by gently cutting and teasing the morphologically undifferentiated cells using a yellow pipette tip and transferring undifferentiated colony pieces to a fresh culture dish. It should be noted that some spontaneous differentiation of the cells was observed in the early passage cultures in absence of FGF2, which is to be expected due to the stress of the altered culture conditions.

### 5.3. Differentiation of hES Cells

Control and neutralizing antibodies were removed from the cultures and the cells differentiated by overgrowth o in DMEM + F12 mix media supplemented with L-glutamine (1 mM), 2-mercaptoethanol (50 μM), NEAA (100×, 1:100 dilution), and bFGF (0.2 μg/mL in 0.1% BSA) (all Invitrogen) without removal of the feeder layer.

### 5.4. Fluorescent Flow Cytometry Analysis of ES Cells

Human ES cells were trypsinized, washed once in 900 μL of PBS and resuspended in 100 μL of 0.2% BSA in PBS (FACS buffer) containing the primary antibody. Primary antibodies were as follows: Tra-1-60 (phycoerythrin conjugated anti-Tra-1-60; Santa Cruz) and incubated for 1 h on ice. Cells were washed once in 900 μL of PBS, resuspended in 100 μL of FACS buffer containing a phycoerythrin-conjugated secondary antibody that recognized the primary antibody (all 1:100 dilution; Santa Cruz) and incubated for 30 min on ice. The cells were washed once in 900 μL of PBS and fixed in 400 μL of 1% formaldehyde. Cell fluorescence was analyzed using a Becton Dickinson FACScaliber. Viable cells were gated using forward and side scatter and the data represent cells from this population.

### 5.5. RT-PCR

Total RNA was extracted from cells using RNAzol B according to the manufacturer's instructions (Biogenesis, Dorset, UK), treated with DNase (Promega, WI, USA) and phenol/chloroform extracted. Synthesis of cDNA from mRNA transcripts was performed using the following method: RNA (10 μg), dNTP (250 μM), oligo (dT) (5.0 μg total), reverse transcriptase (40 U) in a total volume of 200 μL and incubated at 42 °C for 1 h. RT-PCR was performed using 1 μL of the cDNA solution and 35 cycles. Samples were run on 2% agarose gels containing 400 ng/mL ethidium bromide and visualized using an Epi Chemi II Darkroom and Sensicam imager with Labworks 4 software (UVP, CA, USA). Primers used were as follows (read 5′ to 3′; forward-F, reverse-R; all 60 °C annealing): **β-Tub**—**F** GGAACATAGCCGTAAACTGC, **R** TCACTGTGCCTGAACTTACC, 317 bp; **Oct-4**—**F** AGAAGGAGCTAGAACAGTTTGC, **R** CGGTTACAGAACCATACTCG, 415 bp; **AFP**—**F** CCA TGT ACA TGA GCA CTG TTG, **R** CTCCAA TAA CTC CTG GTA TCC, 338 bp; **HNF**—**F** GAG TTT ACAGGC TTG TGG CA, **R** GAG GGC AAT TCC TGA GGA TT, 390 bp; **NES**—**F** GCC CTG ACC ACT CCA GTT TA, **R** GGA GTC CTG GAT TTC CTT CC, 199 bp; **NFM**—**F** GAG CGC AAA GAC TAC CTG AAG A, **R** CAG CGA TTT CTA TAT CCA GAG CC, 430 bp; **NSE**—**F** CCCACT GAT CCT TCC CGA TAC AT, **R** CCG ATC TGG TTG ACC TTGAGC A, 254 bp; PAX—F AAC AGA CAC AGC CCT CAC AAA CA, R CGG GAA CTT GAA CTG GAA CTG AC, 275 bp; PLP—F CCA TGC CTT CCA GTA TGT CAT C, R GTG GTC CAG GTG TTG AAG TAA ATG T, 354 bp (plp) and 249 bp (dm-20); Amy—F GCT GGG CTC AGT ATT CCC CAA ATA C, R GAC GAC AAT CTC TGA CCT GAGTAG C, 490 bp; TRP—F AGA CCC TTT GAA GTC AAG GAC ACCG, R CCA TTG CTG AAG ACC TTA GTG ATG C, 360 bp; Flk-1—F GGT ATT GGC AGT TGG AGG AA, R ACA TTT GCC GCT TGG ATA AC, 203 bp; CD34—F TGA AGC CTA GCC TGT CAC CT, R CGC ACA GCT GGA GGT CTT AT, 200 bp; AC133—F CAG TCT GAC CAG CGT GAA AA, R GGC CAT CCA AAT CTG TCC TA, 199 bp; Tf—F CTG ACC TCA CCT GGG ACA AT, R CCA TCA AGG; R CCA TCA AGG 307 CAC AGC, 367 bp; Oct-4 (OCT); α-fetoprotein (AFP); hepatocyte nuclear factor (HNF); nestin (NES); neurofilament middle chain (NFM); neuron-specific enolase (NSE); Pax-6 (PAX); proteolipid protein (PLP); amylase (AMY); α1-antitrypsin (TRP); Flk-1 (Flk); AC133 (AC1); Transferrin (Tf); β-tubulin (β-Tub); alpha-foetal protein (AFP).

## 6. Conclusions

E-cadherin is emerging as a key regulator of human and mouse stem cell pluripotency and self-renewal. In mouse ES cells, for example, at least two pluripotent pathways exist which are dependent upon E-cadherin protein expression levels, and we have shown that this may also be true in human ES cells (Figure 6). Whilst expression of the core pluripotency genes, Oct3/4, Sox2 and Nanog, appear to be unaltered in wt and E-cadherin^−/−^ ES cells, the latter are capable of self-renewal via LIF/BMP or FGF/Activin/Nodal signaling pathways, suggesting that these cells exhibit at least two independent pathways to maintain pluripotency. Indeed, we have demonstrated reversible Activin/Nodal-dependent pluripotency in wild-type mES cells treated with the CHAVC peptide, suggesting that these cells also exhibit at least two pluripotent signaling pathways. Whilst ES cell “ground state” pluripotency (described by Ying and colleagues [63]) is an interesting concept, in practice it is difficult to uncouple ES cell behavior from the culture environment. Whilst the exact mechanisms controlling the switch between LIF/BMP and Activin/Nodal pathways are not fully understood, it is clear that E-cadherin functions to maintain the hierarchy of these independent pathways.

Cell-ECM interaction has been shown to be important for survival of dissociated pluripotent cells whilst E-cadherin-mediated cell-cell contact appears critical to pluripotent pathway regulation in both mouse and human cells. Since E-cadherin is a cell surface protein it may provide a useful target for the manipulation of culture conditions of pluripotent cells using exogenous compounds. We have previously described a cyclic peptide, CHAVC, which can alter pluripotent signaling pathways in mouse ES cells [87]. However, this peptide is unstable (due to disulphide bonds) and its use is further complicated by having to screen serum batches for optimal activity of the peptide. Therefore, the various small molecules described in this review that can enhance E-cadherin function or antagonise E-cadherin-mediated pluripotency pathways may be useful for manipulating culture conditions for optimal and cost-effective self-renewal of these cells. There appears to be some confusion in the literature as to which pathways are *essential* for pluripotency and which pathways simply enhance the pluripotent state of stem cells. One example of this confusion is that of β-catenin and activation of the Wnt pathway. Knock-out studies have revealed that β-catenin is not required for establishment of the ICM and epiblast, therefore, it can be concluded that this protein is not required for maintenance of pluripotency in a physiological setting. However, it is clear that manipulation of β-catenin activity can enhance stem cell pluripotency *in vitro.* In the context of clinical therapies these points are merely academic and non-physiological pathways that can enhance pluripotency/differentiation of stem cells will be of significant relevance, particularly where associated with increased cost-effectiveness.

Whilst it would be satisfying for us to conclude that E-cadherin is the critical component in regulating pluripotent pathways in ES and iPS cells, this may not be correct. Microarray analysis of E-cadherin^−/−^ ES cells in our lab has revealed over 2000 gene transcript alterations compared to wtES cells. Interestingly, these changes were not restricted to adhesion-related genes but also to transcripts associated with a wide range of cellular processes (e.g., primary metabolic processes, catabolism and apoptosis). When viewed in the context of altered plasma membrane protein localization, it is clear that E-cadherin plays a major role in cellular homeostasis and that abrogation of this protein has a significant impact upon many cellular processes. Many of these changes are likely to be non-specific effects related to loss of epithelial integrity rather than direct regulation by E-cadherin. Therefore, the challenge for the near future is to elucidate and dissect processes associated with E-cadherin expression to allow delineation of pathways which are impacted directly by E-cadherin and those associated with non-specific events due to loss of epithelial integrity. However, the fact that E-cadherin has risen above its previous label of a mere “cell adhesion protein” should be celebrated and future work is likely to realize more unexpected functions of this remarkable protein.
